# Mrs. Mary Alice Frost

**Published:** 1918-02

**Authors:** 


### OBITUARY

---

**Mrs. Mary Alice Frost,** nee Laverack, widow of Dr. Henry C. Frost of Buffalo, died Dec. 23.

---

**Dr. Andrew Taylor Still,** the founder of osteopathy, died Dec. 13, aged 89. Born in Philadelphia, he became a resident of Kansas before the Civil War, was elected to the legislature in 1859 on an anti-slavery platform and served in the war as

